# Hydroxytyrosol in Foods: Analysis, Food Sources, EU Dietary Intake, and Potential Uses

**DOI:** 10.3390/foods11152355

**Published:** 2022-08-06

**Authors:** Marta Gallardo-Fernández, Marina Gonzalez-Ramirez, Ana B. Cerezo, Ana M. Troncoso, M. Carmen Garcia-Parrilla

**Affiliations:** Departamento de Nutrición, Bromatología, Toxicología y Medicina Legal, Facultad de Farmacia, Universidad de Sevilla, C/ P. García González nº 2, 41012 Sevilla, Spain

**Keywords:** hydroxytyrosol, virgin olive oil, wine, olives, dietary intake, food consumption

## Abstract

Hydroxytyrosol (HT) is a phenolic compound with proven biological properties present in a limited number of foods such as table olives, virgin olive oil (VOO) and wines. The present work aims to evaluate the dietary intake of HT in the European (EU) population by compiling scattered literature data on its concentration in foods. The consumption of the involved foods was estimated based on the EFSA Comprehensive European Food Consumption Database. The updated average contents of HT are as follows: 629.1, 5.2 and 2.1 µg/g for olives, olive oil and wine, respectively. The HT estimated intake in the European Union (EU) adult population falls within 0.13–6.82 mg/day/person, with table olives and wine being the main contributors. The estimated mean dietary intake of HT in EU countries is 1.97 ± 2.62 mg/day. Greece showed the highest HT intake (6.82 mg/day), while Austria presented the lowest (0.13 mg/day). Moreover, HT is an authorized novel food ingredient in the EU that can be added to different foods. Since the estimated HT intake is substantially low, the use of HT as a food ingredient seems feasible. This opens new possibilities for revalorizing waste products from olive oil and olive production which are rich HT sources.

## 1. Introduction

**Figure 1:**
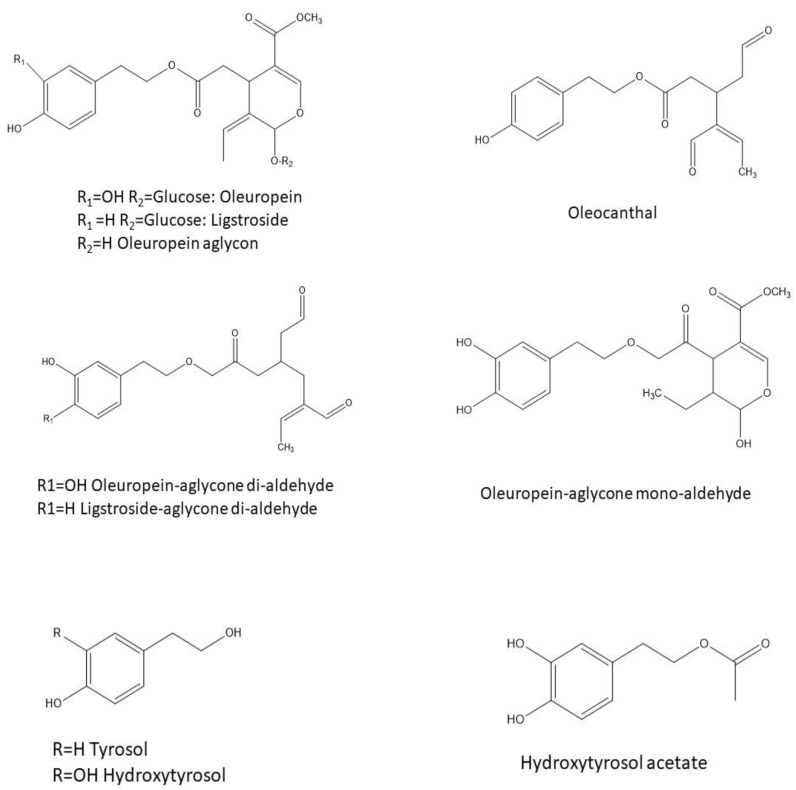
Structures of the main hydroxytyrosol-related compounds described in olives and olive oil.

A Mediterranean diet has been associated with a decreased incidence of cancer, cardiovascular and neurodegenerative disorders. Since epidemiological data associate the consumption of certain foods such as olive oil, nuts, fruits and vegetables to a lesser incidence of disease, intense research is conducted to explain how the compounds with biological properties (bioactives) might exert their action. In particular, polyphenolic compounds abundant in plant foods have been extensively studied. This is the case for hydroxytyrosol (HT), present mainly in olives, virgin olive oil and wine, which has attracted scientific interest due to its ortho-dihydroxy conformation in the aromatic ring, a chemical feature related to its antioxidant bioactivity. Indeed, Villaño et al. [1] highlighted that the dihydroxy substitution in the ortho position was related to a higher antioxidant activity when comparing different phenolic compounds (Figure 1). At the same time, Napolitano et al. [2] showed that HT possesses a better H-donor than another simple phenol such as tyrosol (TYR) as well as a greater iron-chelating ability. Thus, HT is more efficient as antioxidant than TYR. HT presents the strongest antioxidant activity among the major olive phenols [3]. Furthermore, its antioxidant effect is not solely due to the capacity of scavenging oxidant chemical species, but to the ability of stimulating the activity and synthesis of antioxidant enzymes [4]. Additionally, HT has shown anti-inflammatory, neuroprotective and anti-angiogenic effects [5,6,7,8,9,10,11,12,13,14,15].

All in all, recent literature is gathering evidence that HT may have an important role protecting the most prevalent non-infectious diseases, which are also related to diet. However, it remains difficult to establish the current dietary intake of HT. For this purpose, the first task is to estimate the occurrence of this bioactive compound in foods. With olives, olive oil and wine as the main dietary sources, there are factors to consider such as origin, variety, maturity, food processing, and storage, among others. In this sense, intense efforts have been made to compile food composition data regarding bioactives, HT among them. For example, Phenol Explorer [16] is a database that summarizes scattered data on the phenolic composition of foods in scientific literature. This has proved challenging due to the overwhelming number of papers published each year and the incorporation of new food data since the last Phenol Explorer update (2015). In terms of HT literature, access to Scopus in December 2021 showed a growing trend in the number of articles: 30 papers in 1991, 118 in 2001, and more than 334 in 2021, including different perspectives and scopes.

In regard to food composition data, reliability clearly depends on the analytical methods used to obtain the reported values, thus special attention needs to be paid to the description of these methods in the literature.

In summary, the aim of this work is to evaluate HT dietary intake for the adult population in Europe and to ascertain if its addition to foods as a bioactive ingredient is feasible in the future. In this context, it is important to ensure that the sum of the amount naturally provided by foods and the amount provided as a food ingredient is below any toxic levels, due to safety concerns.

## 2. Methodology

### 2.1. Literature Search

A scientific literature search was conducted using the Scopus database until July 2022. The initial search with HT as a keyword was further refined with the food source (Table 1, Table 2 and Table 3). We selected the articles including the analytical determination of HT, TYR, and secoiridoids (oleuropein, oleuropein aglycone, and elenolic acid dialdehydes), since they are HT precursors.

### 2.2. Estimation of Hydroxytyrosol Dietary Intake in Europe

The 2011 EFSA survey, namely the Comprehensive European Food Consumption Database was used [59] to estimate the mean consumption of olive oil, table olives and wine per person in the different European countries, following the previously reported methodology for the intake estimation of other polyphenolic compounds [60]. The food consumption of twenty-two different countries was considered (Austria, Belgium, Croatia, Czech Republic, Cyprus, Denmark, Estonia, Finland, France, Germany, Greece, Hungary, Ireland, Italy, Latvia, Netherlands, Portugal, Romania, Slovenia, Spain, Sweden and the United Kingdom) since the other countries either did not present data or presented data that did not refer to the adult population. Data regarding the most recent surveys were considered (Table 4). For each food items, the mean consumption data in g/day per person was used, taking into account that all subjects in the surveys were included, not only those consuming the foods under study.

Concerning food composition, concentration in each food was expressed in ng/g. In the case of wine, the corresponding density was used to convert the intake to ng/g. Finally, HT dietary intake in each country was expressed as the sum of the contribution of all the foods under study in mg/day/person. Statistical analyses were performed using STATISTICA 7^®^ (Palo Alto, CA, USA).

## 3. HT Health Benefits and Mechanisms of Action

HT may act in vivo as a strong anti-inflammatory agent, inhibiting lipopolysaccharide (LPS)-mediated expression of inflammatory cytokines, i.e., TNF-α and IL-1β [5]. Indeed, these effects are also related to a neuroprotective role. Microglial activation, expression of NADPH oxidase and MAPKs, production of ROS, and activation of the inflammasome induced by LPS were reduced or prevented by HT. HT was also able to decrease the activation of microglial cells after alpha-synuclein (α-syn) treatment [6], thus demonstrating its capacity to reduce neuroinflammation. Furthermore, it is important to highlight its neuroprotective effect against Parkinson and Alzheimer’s diseases, being able to inhibit the formation of α-synuclein [7,8] and β-amyloid fibrils [9], respectively.

Furthermore, HT exerts antiangiogenic effect by VEGF receptor-2 (VEGFR-2) inhibition [10]. Angiogenesis in adults is involved in the development of cancer and cardiovascular diseases, favoring both tumor development and the development and destabilization of the atheroma plaque [11]. Indeed, tumors express various pro-angiogenic factors, the main one being VEGF, which binds to VEGFR-2 [12]. In addition, HT decreases proliferation in MCF-7 (Michigan Cancer Foundation-7) breast cancer cell model [13]. This effect may be linked to its pro-apoptotic activity [14].

In addition, several studies indicate that HT has an insulin-like effect on target cells including adipocytes, hepatocytes and muscle cells, exerting significant anti-diabetic effects in animals’ models of type 2 diabetes mellitus [61,62].

Certainly, its best-known biological effect is related to blood lipids’ protection from oxidative stress. [15]. In particular, the EU authorized health claim states that “Olive oil polyphenols contribute to the protection of blood lipids from oxidative stress”, based on the protection of LDL particles from oxidative damage. The health claim can be applied to olive oils containing at least 5 mg of HT and its derivatives (e.g., oleuropein complex and TYR) per 20 g of olive oil. Table 5 summarizes the HT mechanisms of action.

## 4. Determination of HT in Foods

In order to evaluate dietary intake, it is crucial to select reliable data on food composition that largely depends on the performance of the analytical determination. Therefore, it is useful to understand the advantages and drawbacks of the analytical methods used and the possible matrix effect and interferences. HT is present as a minor compound within a complex food matrix, and it is generally determined simultaneously with the phenolic profile. In fact, regarding olive oil, the official method of the International Olive Council (IOC) [63] quantifies HT together with TYR, natural and oxidized oleuropein and ligstroside derivatives, lignans, flavonoids and phenolic acids in olive oil, overall expressed as biophenols.

The first step and most critical point when analyzing HT in olive oil is its extraction from the lipidic food matrix. Solid phase extraction and liquid–liquid (methanol:water 80:20) extraction have been used [64] to extract the polar fraction that contains HT. This last method, which performs acidic hydrolysis before extraction, is expected to become an official method.

Other food matrixes required different pre-treatments. Olive fruits are grinded, followed by extraction with methanol, ethanol or mixtures of both with water. Then, n-hexane is the solvent most frequently used to purify the extract. Optionally, freeze-drying can be performed [65]. In some cases, the extracts are loaded onto SPE cartridges with hexane followed by methanol to eluate HT. For the wine matrix analysis, direct injection of the sample was used with high performance liquid chromatography (HPLC) coupled to fluorescence detectors [58]. Álvarez-Fernández et al. [53] proposed cleaning up the sample with C18 SPE cartridges followed by elution with methanol for subsequent determination by ultrahigh performance liquid chromatography couple to high-resolution mass spectrometry (UHPLC/HRMS). Additionally, the wine extract can be treated after purification with an Amberlite column and separated by high-speed counter current chromatography prior quantification by DAD or HRMS [66].

Liquid chromatography is the most widespread and reliable method for analyzing HT individually in the different foods as displayed in Table 1, Table 2 and Table 3. Reversed-phase, which is based on partition chromatography, is the preferred mode of separation for most polar compounds such as HT in terms of reproducibility of retention time and separation [66]. It separates individual components using a non-polar octade-cylsilane (C18) column as the bonded stationary phase, while the mobile phase is a polar solvent consisting of water acidified with orthophosphoric acid, formic acid, acetic acid, or trichloroacetic acid, and methanol or acetonitrile [27,33,53]. Different detection techniques include the use of a diode array (DAD) detector at 280 nm since phenols possess a strong chromophore system, providing considerable structural information that helps distinguish the type of phenol and the oxidation pattern [67]. Additionally, a fluorescence detector has also been used since HT shows a good response to fluorescence excitation (λex = 279 nm and λem = 631 nm) [68]. However, the most extended powerful analytical tool is the liquid chromatography coupled with mass spectrometry (LC-MS) as a detection system, which provides unequivocal identification based on the molecular masses of the separated compounds obtained through prominent ions [69]. The limit of detection (LOD) and quantitation (LOQ) differ among the techniques being up to 0.09 mg/L and 0.3 mg/L for DAD, and 0.023 mg/L and 0.076 mg/L for fluorescence, [52,58] and 0.5 µg/L and 1 µg/L for LC-MS/MS, respectively [17].

## 5. Dietary Sources

Despite being a phenolic compound, HT is present in limited food sources. HT has been identified in foods characteristic of the Mediterranean diet such as table olives, olive oil and wine (Table 1, Table 2 and Table 3). HT and its derivates compounds (e.g., oleuro-pein complex and tyrosol, as defined by EFSA [15]) have been only reported in table olives and olive oil (Table 1 and Table 2). Additionally, HT is synthesized by yeast during the alcoholic fermentation and found consequently in wine (Table 3). Solely one study has reported HT in commercial beers at a concentration (ca. 0.03 mg/L) significantly lower than the abovementioned dietary sources [70].

### 5.1. Olives

Up to 36 phenolic compounds have been identified in olive fruits, with secoiridoids being the most abundant and widespread (Figure 1). Among them, oleuropein and ligstroside are the major main native compounds [71]. Their breakdown products include relevant phenolic constituents of the olive fruit such as HT, oleocanthal, elenolic acid, oleuropein aglycone, and TYR [71]. During olive ripening, storage, and processing, hydrolysis of the secoiridoid compounds yields HT [72]. Generally, as oleuropein decreases, HT increases during maturation, making this substance the main compound in mature olives.

Most table olives worldwide are processed using one of three methods: Spanish-style (green olives), Californian-style (olives darkened by oxidation), or Greek-style (natural darkened olives) processing. Spanish-style includes lye treatment, brining and fermentation. Lye treatment with a NaOH solution (1.5–4.5 *w/v*) causes the hydrolysis of oleuropein to yield HT and elenolic acid glucoside [73]. Furthermore, yeasts and lactic acid bacteria, naturally present in olives and brine, can metabolize oleuropein in a two-step process. The first step comprises the hydrolysis of the glycosidic linkage of oleuropein by β-glucosidase to form oleuropein-aglycone. Subsequently, this aglycone is hydrolyzed to elenolic acid and HT, probably by an esterase in a second step.

Californian-style black olive processing consists of preserving the fruits in brine or an acidified solution followed by darkening with air under alkaline conditions. The first step causes the diffusion of polyphenols, mainly oleuropein, from the olive flesh into the solution and acid hydrolysis takes place as previously mentioned. Subsequently, the darkening step basically causes ortho-diphenols to be oxidized and polymerized resulting in a decrease in HT. After harvesting, the olives are placed in brine where they are fermented. Similar to the other described processes, the acid hydrolysis of oleuropein and HT glucoside occurs.

Table 1 summarizes data published in the literature on HT and derived compounds present in table olives. HT concentration reported by different authors using the above-mentioned analytical methods does not reflect significant differences (*p* > 0.05) between Spanish and Greek table olives. Values range from 14.49 to 3750 mg/kg and from 134.33 to 1393.30 mg/kg for Spanish and Greek style, respectively. Values for Californian style olives are in general lower, ranging from 19.98 to 672.40 mg/kg. This observation is in agreement with the results reported by Johnson et al. [27], whose predominant phenolic compound was HT in all three styles of commercial olives with similar concentrations observed for Greek and Spanish olives (134.329 and 133.685 mg/kg, respectively) and significantly lower concentrations for Californian olives (19.981 mg/kg). Romero et al. [74] detected the presence of hydroxytyrosol 4-β-D-glucoside in the olive pulp of the Manzanilla and Picual varieties, pinpointing that not only the hydrolysis of oleuropein produces HT, but it can also come from hydroxytyrosol 4-β-D-glucoside hydrolysis. Indeed, Arroyo López et al. [75] found hydroxytyrosol 4-β-D-glucoside content was greater than that of oleuropein.

As can be seen in Table 1, HT is the main phenolic compound in edible olives followed by oleuropein and TYR. In summary, the processing method is the most determinant factor compared to the variety or geographical origin.

### 5.2. Olive Oil

Table 2 summarizes the concentration of HT, TYR, hydroxytyrosol acetate (HT-AC), oleuropein-aglycone di-aldehyde (3,4-DHPEA-EDA), oleuropein-aglycone mono-aldehyde (3,4-DHPEA-EA), ligstroside-aglycone di-aldehyde (p-HPEA-EDA), oleuropein (OLE), and secoiridoid derivatives (S-DER) (Figure 1) reported in olive oils from different origins, varieties and categories. As can be seen, the maximum and minimum concentration of HT determined are between 41.3 mg/kg and 0.09 mg/kg, respectively, with noticeable differences according to origin. In particular, the highest mean HT concentration is found in olive oils from Spain (13.31 ± 39.45 mg/kg), followed by Greece (11.17 ± 8.22 mg/kg), and Italy (10.24 ± 29.09 mg/kg). Samples from other origins, such as Tunisia, Algeria, Morocco, and Turkey, present lower concentrations as follows: 5.51 ± 2.36 mg/kg, 3.86 ± 2.73 mg/kg, 3.29 ± 2.44 mg/kg, and 1.89 ± 2.39 mg/kg, respectively.

**Figure 2:**
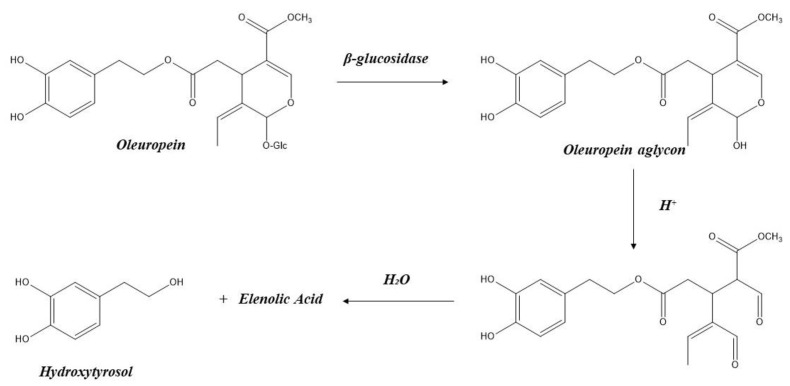
Hydroxytyrosol formation pathway from oleuropein in olive oil.

Some analytical procedures include an acid hydrolysis that allows the determination of not only free HT content in olive oil but also the resultant from oleuropein hydrolytic degradation [76] (Figure 2); these values reach 151.5 mg/kg for an oil of Italian origin and 200 mg/kg for an olive oil of Spanish origin. Hence, these values were not considered for the dietary HT intake estimation. It is difficult to ascertain the actual concentrations of oleuropein and HT since different values will be obtained depending on the storage time of the oil prior to analysis. Therefore, as the storage time increases, the concentration of oleuropein decreases and that of HT increases [77]. When we applied analysis of variance (ANOVA) to the HT data displayed in Table 3, no significant differences were observed between the different varieties or between VOO and EVOO (extra virgin olive oil).

In addition, secoiridoids are hydrolyzed during oil storage, giving rise to the simple phenolic compounds HT and TYR [77]. In fact, HT and TYR concentrations increased after one year of storage in three olive oil varieties, in the following order from highest to lowest concentration: Picual > Hojiblanca > Arbequina [77]. A recent study [78] confirms those findings, observing very low HT (4.72 µg/g of EVOO) and tyrosol (5.47 µg/g of EVOO) concentrations in fresh EVOOs and higher concentrations in one-year old EVOO (20.18 µg/g of EVOO and 54.51 µg/g of EVOO, respectively). Thus, we can conclude that fresh EVOO has a high content of oleocanthal and oleacein and a low concentration of tyrosol and hydroxytyrosol; the hydrolytic processes during the storage time led to the formation of tyrosol and hydroxytyrosol, from their precursors.

Apart from storage time, it must be considered that olive oil is also consumed after heating such as frying, boiling, and oven cooking, which substantially impact the concentration of phenolic compounds to a different degree, depending on the initial concentration and treatment characteristics (time, temperature, humidity). HT derivatives are the first antioxidants lost during thermal oxidation (up to a peroxide value of 20–30 mEq/kg), and TYR derivatives seem to be the most stable compounds [79].

### 5.3. Wine

Di Tommaso, Calabrese, & Rotilio [57] found HT for the first time in wines at concentrations ranging from 1.9 mg/L to 4.0 mg/L. Later, HT was identified in different wines worldwide varying from 1.5 to 41.5 mg/L as displayed in Table 3. As can be observed in this table, a high percentage of references (around 83%) report an HT concentration of between 1–4 mg/L, regardless the type of wine, origin or variety. In contrast to olives’ composition, TYR is frequently present in wines at higher concentrations than HT, as displayed in Table 3.

**Figure 3:**
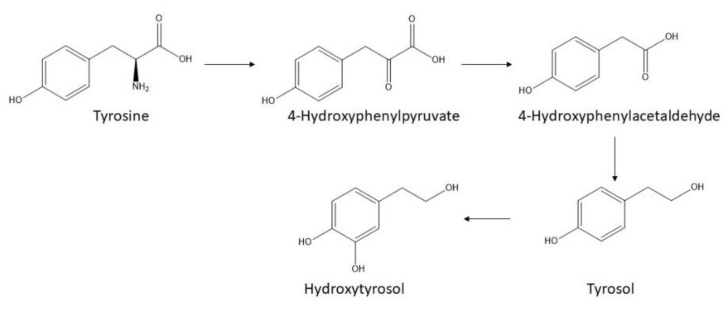
Illustration of the biosynthetic pathway of hydroxytyrosol (3,4-dihydroxyphenethyl alcohol, DOPET, 3-hydroxytyrosol, and homoprotocatechuyl alcohol. The empirical formula of hydroxytyrosol is C_8_H_10_O_3_ and it has a molecular weight of 154.16 g/mol) from tyrosine for different organisms.

HT occurrence in wine can be related to yeasts’ metabolism. In fact, during alcoholic fermentation, the yeasts metabolize aromatic amino acids by the Ehrlich pathway. As Figure 3 shows, tyrosine can be transformed into p-hydroxyphenylpyruvate by a transamination reaction. Subsequently, decarboxylation of this last compound produces p-hydroxyphenylacetaldehyde, which can be metabolized to form the fusel alcohol [80]. Finally, a tyrosine hydroxylase can generate HT from TYR. Álvarez-Fernández et al. [53] identified HT in the intracellular compartment of wine-making yeast both in *Saccharomyces cerevisiae* strains and non-*Saccharomyces Torulaspora delbrueckii*, providing unequivocal proof of the yeast’s role.

In both white and red wines, HT has also been detected at concentrations ranging from 0.28 to 9.6 mg/L. As Table 3 reports, the concentrations determined by different authors do not show a clear difference between white and red wines. Furthermore, no conclusion can be inferred regarding the variety, origin, aging or year of the wine.

## 6. Estimation of HT Dietary Intake

Different strategies have been proposed to evaluate the dietary intake of micronutrients or bioactive compounds. For most nutrients and bioactives, dietary surveys of food intake, questionnaires, and the use of food composition databases to transform food consumption into nutrient intake can be considered a suitable approach [60].

In this context, the determination of HT in urine has been associated with the intake of foods rich in this phenolic compound such as olives, olive oil, and wine [81]. In particular, it could be proposed as a marker to monitor compliance in the consumption of dietary extra virgin olive oil [27]. Nevertheless, Schröder et al. [82] reported higher urinary recovery of HT than expected after red wine consumption. This fact led researchers to hypothesize that an endogenous synthesis of HT may occur. Thereafter, human intervention studies confirmed higher HT sulfate metabolism after alcohol consumption [83] in a dose-dependent manner. Furthermore, excretion after wine consumption was higher than excretion after alcohol or dealcoholized wine [84]. Considering these data as a whole, it is clear that HT is not only a compound present in foods, but also a human metabolite related to tyramine and dopamine metabolism [85].

Several studies have demonstrated that TYR is converted to HT in vivo [86]. Therefore, these findings imply that TYR intake has to be taken into account when discussing circulating HT levels. Thus, urinary excretion of HT might not be an accurate marker of dietary HT intake. Consequently, another approach should be considered based on the available scientific available data on food composition and food intake.

Table 6 estimates the daily intake of free HT from the consumption of the foods under study (range of means) based on the reported mean free HT content in extra-virgin olive oils (5.2 µg/g), table olives (629.1 µg/g) and wine (2.1 µg/mL) (Table 1, Table 2 and Table 3) and their estimated intake in the EU, as reported in the EFSA Comprehensive Food Consumption Database [59]. These data are based on surveys of more than 45,000 participants (Table 4). Although HT has been reported in beer, it has not been included in the estimation of HT dietary intake given the limited information so far available [70].

**Figure 4:**
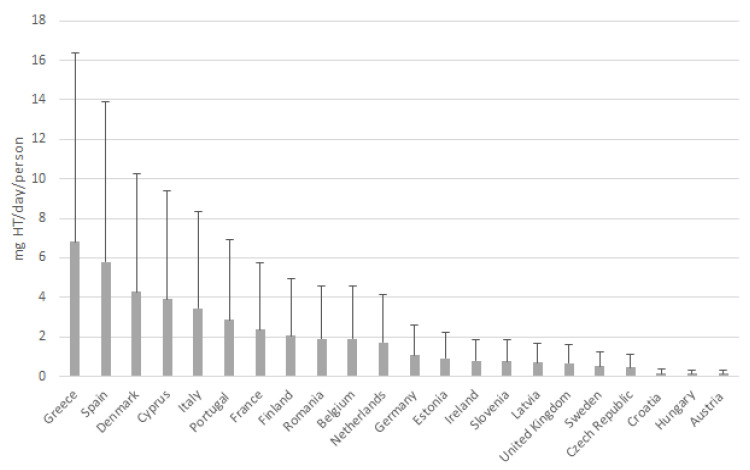
Estimation of the mean free HT dietary intake in the EU adult population.

Figure 4 shows the estimation of the mean dietary intake of free HT in the adult population in each of the twenty-two European countries under study. The HT intake in the EU is within the range of 0.13–6.82 mg/day. The country with the highest free HT intake was Greece (6.82 mg/day), followed by Spain (5.79 mg/day), Denmark (4.28 mg/day), Cyprus (3.90 mg/day) and Italy (3.46 mg/day). In contrast, Austria, Hungary, and Croatia showed the lowest intake (0.13, 0.13, and 0.14 mg/day, respectively). The HT intake of the rest of the countries was within 0.46–3.90 mg/day. The estimated mean HT dietary intake in EU countries is 1.97 ± 2.62 mg/day. Only Greece and Spain have mean values that reach an adequate amount to exert beneficial cardiovascular effects of 5 mg/day [15].

**Figure 5:**
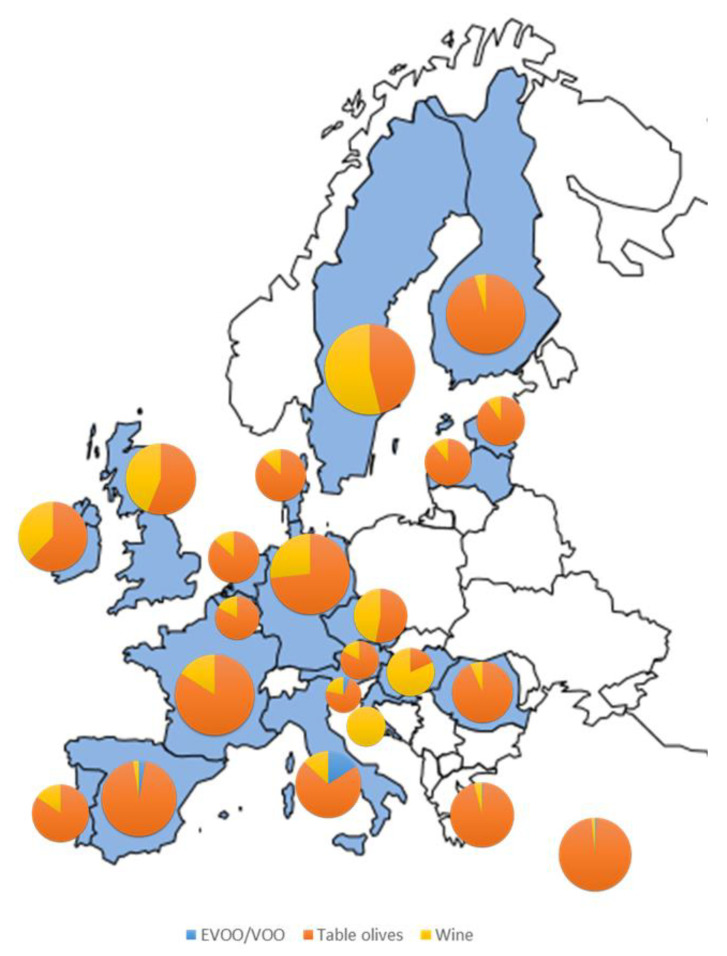
Relative contribution of EVOO/VOO, olives, and wine to free HT dietary intake in the European adult population by country (percentage %).

Figure 5 presents the contribution of the different foods under study to HT intake in the different EU countries. As can be seen, table olives and wine are the main contributors to the HT dietary intake in UE countries. Specifically, olives are the principal HT dietary source in all the countries except for Croatia, Hungary, and Sweden, where wine is the biggest contributor one. Although the amount of table olives effectively consumed is not especially relevant, their HT content is the highest among foods. Therefore, we may assume that EU adults that do not consume wine or olives will not consume HT in their diets. Surprisingly, EVOO/VOO significantly contributes to free HT intake only in some of the Mediterranean basin countries (Italy, Spain, Cyprus, and Greece) and in Slovenia (16%, 3%, 0.7%, 0.6% and 6% of the overall HT dietary intake, respectively).

The estimation of HT intake was based on free HT. However, EVOO/VOO and table olives also contain oleuropein and oleuropein-aglycone, which can be hydrolyzed to HT as it is bioavailable, thus contributing to total HT intake [87]. Therefore, the actual total dietary HT intake may be higher than the estimated exposure based solely on free HT. Accordingly, the EFSA Scientific Panel assessed that total HT exposure from olives and EVOO/VOO may be estimated to be approximately three and six times higher, respectively, than the exclusive exposure to free HT from these foods [88]. Taking into account these values, total HT intake from EVOO/VOO and table olives would range between 0 and 1.08 mg/day and between 0.03 and 6.42 mg/day, respectively. Therefore, the contribution of EVOO/VOO to total HT intake in the Mediterranean basin countries and in Slovenia would be approximately two times higher (30% in Italy, 6% in Spain, 1% in Cyprus, 1% in Greece, and 13% in Slovenia). Additionally, we should consider the fact that some surveys underestimate EVOO/VOO consumption since they include all the olive oils as one food item and no differentiation is made by the subjects. Moreover, thermal oxidation of EVOO/VOO during frying (180 °C) for 30–60 min causes HT losses between 60% and 90% [89], which in turn would overestimate the HT intake from EVOO/VOO when used for frying.

The EFSA Scientific Panel estimated the intake of dietary HT in the EU population just based solely on the contribution of olive oils and table olives as main food sources [88]. Additionally, the EFSA Panel used the Phenol-Explored database, which was developed with the literature available until 2009, to calculate the mean HT content in these foods (0.007 mg/g in EVOO/VOO and 0.65 mg/g in table olives). However, the present study includes current literature published up to 2022. Additionally, our data show for the first time that wine is one of the main contributors, together with olives, to the dietary HT intake in the EU population. Therefore, wine consumption should be taken into account for the assessment of dietary exposure to this compound to avoid its underestimation.

Total dietary polyphenol intake in the adult population of EU countries has been reported to be between 1706 mg/day (Denmark) and 664 mg/day (Greece) [90]. Considering the mean HT intake for these countries, the relative contribution of the single compound HT to the total polyphenol intake would be around 0.2–1%, respectively. Mean HT intake (1.9 mg/day) is within the magnitude order of the intake of certain individual anthocyanins, such as peonidin and petunidin (1.6 mg/day and 1.13 mg/day, respectively) [91], certain flavonols, such as isorhamnetin (2 mg/day), the flavanone eriodictyol (1 mg/day), and the flavone luteolin (1 mg/day) estimated for the EU population [60].

## 7. Hydroxytyrosol as a Food Ingredient and Future Trends

The EFSA delivered a positive opinion on the health claim “Olive oil polyphenols contribute to the protection of blood lipids from oxidative stress” for extra virgin olive oil (20 g) containing 5 mg of HT and its derivatives [15]. Moreover, HT is recognized as safe by the European Food Safety Authority [88] and, consequently, the European Commission (2017) [92] authorized the placement of HT in the market as a novel food ingredient under Regulation (EC) Nº 258/97. The food categories authorized for HT addition are fish and vegetable oils (0.215 g/kg) and spreadable fats (0.175 g/kg). According to the dietary data from the EU [88], it is estimated that the mean values for HT consumption in adults are far from the toxicity level, which emphasizes the suitability of the incorporation of HT into other types of products.

The antioxidant and antimicrobial character of HT as well as its bioactive potential is directing the use of HT as a food ingredient. At present, this strategy is almost restricted to the scientific field; however, the promising results obtained may consolidate the addition of HT to foods as a trend in the future. Table 7 presents several formulations, including the amount of HT proposed, its origin, and the food category subject to addition such as yogurts, cookies, blood orange juice and smoothies, increasing the bioactive potential of a diet. Additionally, it may be added to meat-derived products to prevent oxidation in line with consumers’ preferences for more natural ingredients.

HT can be obtained by chemical synthesis, produced by biotechnological procedures, or extracted and purified from wastes from food processing, mainly olive oil production and its by-products such as leaves. Indeed, olive oil residues are a natural source of HT in addition to other phenolic compounds. Consequently, different strategies to recover HT from wastes have been evaluated to select the richest waste source and the most efficient and greenest process. The point is to use a polluting product to obtain a natural food ingredient with healthy properties. Lesage-Meessen et al. [99] determined that the extracts obtained by the two-phase olive oil system were significantly higher in HT (1.4 fold) than those obtained for the three-phase method. Allouche, Fki, & Sayadi, [100] obtained a good yield (85.46%) thanks to ethyl acetate used in the batch, allowing HT to be recovered from three-phase olive processing wastewaters. Microfiltration allowed the recovery of HT, which was the main compound in the permeate, representing 54% of the total polyphenols with a HT concentration of 88.7 mg/L [101]. Furthermore, solid phase extraction was evaluated to compare the capacity of different resins to adsorb HT from wastewaters and solvents to desorb it from the resins [102]. The ENV+ resin was able to adsorb almost all the HT and the acidified ethanol mobilized almost all the polyphenols (including HT) adsorbed onto the resins. In addition, ultrasonication has been suggested to improve recovery [103]. Moreover, tangential membrane filtration has been successfully applied to recover phenols from olive oil mill wastewaters, obtaining high concentrations of HT (up to 7203.7 mg/L in the concentrate) [98]. Additionally, enzymatic hydrolysis to release HT has also been evaluated using a culture in *Aspergillus niger* broth, thus the culture increases the amount of HT obtained [104]. More recently, dried olive mill wastewaters have been obtained using a spray dryer. The resulting phenolic rich dried powder contains, among other phenols, substantial amounts of HT, tyrosol and oleuropein (1.48, 2.04 and 103 mg/kg dry weight, respectively) [105].

A hydrothermal treatment was proposed to obtain HT from solid waste from two-phase olive oil extraction or “alperujo” [106] to provoke an autohydrolysis process which favored subsequent HT extraction. Serrano et al. [107] extracted 1600 mg of HT per 1 kg of olive mill solid waste using a thermal pre-treatment at 170 °C for 60 min. Fernández-Bolaños et al. [108] reported having obtained 4.5–5 kg of HT from approximately 1000 kg of alperujo with 70% humidity, which could be subsequently purified to obtain at least 3 kg of HT at 90–95% of purity.

Moreover, olive leaves represent an agricultural residue that can be used as a source of bioactive compounds [109]. Oleuropein was extracted from the leaves [110] using solid phase extraction. Briante et al. [111] used a thermophilic β-glycosidase immobilized on chitosan that permitted the biotransformation of *Olea europaea* L. leaf extract, obtaining eluates with high amounts of HT. Similarly, a simple hydrolysis reaction allowed a relatively high amount of purified HT to be obtained from *Olea europaea* leaf extract (2.3 g per 100 g of fresh olive leaves) [112]. Herrero et al. [113] optimized pressurized liquid extraction, achieving up to 8.542 mg HT/g dried extract using water as an extracting agent, while oleuropein was present in ethanolic extracts (6.156–2.819 mg/g extract). Finally, HT and derivatives can also be obtained in substantial quantities (16.21 mg HT/L residue) from the wastewater generated from table olives production [114].

Summarizing, the current regulatory status for HT within the EU context as an authorized novel food ingredient that can be added to different foods as stated in the Commission Implementing Decision [92] opens possibilities of broadening its use in other foods in the future. Obtaining HT for further uses as a food ingredient from different wastes and byproducts represents a straightforward opportunity for the revalorization of olive oil cultivars, consequently, leading to a more sustainable production.

## 8. Conclusions

This paper compiles scientific data on HT content in the main food sources. The average content range was as follows: olives > olive oil > wines. The dietary intake in the EU population was estimated based on dietary surveys and updated with current compositional data available in the scientific literature. Greece, Spain, Denmark, Cyprus, and Italy rank as having the largest intake in adult populations. Olives and wine are the main dietary contributors to HT intake in EU. Additionally, the contribution of olive oil to HT dietary intake is relevant in Mediterranean countries (Italy, Spain, Greece, and Cyprus).

Considering HT is present only in food characteristic of the Mediterranean diet, further epidemiological research would be needed to bring to light the role of HT in chronic diseases, other than cardiovascular.

Further research is also necessary to increase the HT concentration in natural sources such as wine, by means of selecting the highest producer microorganisms and fermentation conditions. Additionally, it would be interesting to select the olive production techniques that most preserve HT concentration in the final product, avoiding its losses on the wastewater. Similarly, additional studies should be conducted to select the olive varieties and olive oil production techniques that lead to a higher HT and its precursors’ concentration. Moreover, HT obtained from waste and by-products of the olive industry can be considered a valuable source for HT as a food ingredient to be added to other foods, nutraceuticals, and food supplements, thus increasing the bioactive potential and its dietary intake. Hence, further studies should comprise the selection of efficient and environment friendly HT extraction and purification techniques.

## Figures and Tables

**Table 1:** Oleuropein (OLE), tyrosol (TYR), hydroxytyrosol (HT) and hydroxytyrosol acetate (HT-AC) concentration (mg/kg) in table olive samples according to origin, variety, processing and analytical method.

Origin	Variety	Processing	Concentration (mg/kg)				Method	References
			OLE	TYR	HT	HT-AC		
Spain	Marfil	Greek-style	1.40 ± 0.31	201.2 ± 23.2	384.1 ± 81.2	2.85 ± 0.71	LC-ESI-MS/MS	[17]
	Azeitera	Spanish-style	105.9 ± 4.7	60.2 ± 5.6	605.1 ± 10.6	n.dr.	RP-HPLC-DAD (OLE) RP-HPLC-FLD (TYR, HT)	[18]
			96.8 ± 6.5	54.2 ± 3.9	581.2 ± 12.5	n.dr.		
	Carrasqueña	Spanish-style	150 ± 10.5	75.1 ± 5.2	825.3 ± 14.5	n.dr.		
			138.4 ± 5.9	80.2 ± 4.8	812.5 ± 11.5	n.dr.		
	Conserva de Elvas	Spanish-style	86.4 ± 2.6	35.2 ± 5.6	415.6 ± 8.5	n.dr.		
			80.9 ± 4.7	39.6 ± 2.1	394.1 ± 10.6	n.dr.		
	Morisca	Spanish-style	80.5 ± 6.9	49.8 ± 4.6	398.6 ± 5.8	n.dr.		
			86.9 ± 2.8	45.6 ± 6.4	455.9 ± 7.1	n.dr.		
	Carrasqueña	Spanish-style	3 ± 1	64 ± 10	876 ± 82	n.dr.	RP-HPLC-DAD	[19]
	Manzanilla	Spanish-style	1411.0 ± 452.7	78.6 ± 6.4	1005.5 ± 25.4	n.dr.	HPLC-MS	[20]
	Hojiblanca	Spanish-style	96.3 ± 42.8	79.1 ± 15.4	1133.1 ± 110.6	n.dr.		
		Darkening	n.dr.	19.5 ± 2.1	40.9 ± 6.3	n.dr.		
		Darkening	n.dr.	55 ± 3	275 ± 10	n.dr.	HPLC-DAD	[21]
Italy	Ascolana tenera	Natural-style	553.4 ± 133	97.2 ± 6.5	1770.3 ± 324	n.dr.	RP-HPLC-FLD	[22]
		Spanish-style	209.3 ± 8	60.7 ± 1.0	391.2 ± 8	n.dr.		
			156.3 ± 3.9	63.1 ± 1.4	372 ± 10.1	n.dr.		
			89.2 ± 5.2	38.5 ± 4.3	103.6 ± 8	n.dr.		
			77.9 ± 6.7	29.7 ± 5.4	92.5 ± 11	n.dr.		
			225 ± 2.9	225.5 ± 13	752.5 ± 10	n.dr.		
			79.2 ± 2.1	71.2 ± 4.2	402.1 ± 5.1	n.dr.		
			76.8 ± 6.7	60.4 ± 8.5	324.5 ± 9.4	n.dr.		
	Leccino	Greek-style	0.00	22.78 ± 6.15	150.17 ± 41.20	n.dr.	UHPLC-DAD	[23]
	Bella di Cerignola	Greek-style	n.dr.	80.5 ± 6.9	421.8 ± 30.7	26.7 ± 0.9	HPLC-DAD	[24]
	Termite di Bitetto	Greek-style	n.dr.	28 ± 0.7	258.2 ± 12.1	0.0		
	Cellina di Nardò	Greek-style	n.dr.	353.5 ± 26.2	1393.3 ± 38	0.0		
	Nocellara del Belice	Spanish-style	35.8 ± 10.3	51.5 ± 4.1	535.4 ± 37.2	62.3 ± 11	HPLC-MS	[25]
		Castelvetrano-style	49.6 ± 16.5	49.0 ± 11.7	715.8 ± 79.4	26.8 ± 13.8		
Portugal	Negrinha de Freixo	California-style	n.dr.	161.3 ± 15.5	672.4 ± 75.4	n.dr.	RP-HPLC-DAD	[26]
	Galega	Not mentioned (naturally black)	n.dr.	139.1 ± 24.0	3833.0 ± 180.9	n.dr.		
California USA	Kalamata	Greek-style	7.303	1.315	134.329	n.dr.	UHPLC-(ESI) MS/MS	[27]
	Manzanillo	California-style	0.974	0.435	19.981	n.dr.		
		Spanish-style	3.205	0.859	133.685	n.dr.		
	Manzanilla	California-style	36.7 ± 3.1	n.dr.	210.0 ± 18.8	n.dr.	UHPLC-QqQ MS/MS dMRM	[28]
	Mission	Dry-salted black olives	516.2 ± 44.3	n.dr.	633.8 ± 55.1	n.dr.		
Greece	Throuba Thassos	Dry-salted black olives	1459.5 ± 100.1	n.dr.	195.1 ± 7.8	n.dr.		
Algeria	Azz Sed	Spanish-style	n.dc.	37.27 ± 0.73	105.97 ± 12.2	n.dr.	RP-HPLC-DAD	[29]
	Gordal	Spanish-style	n.dc	43.65 ± 1.09	45.68 ± 1.49	n.dr.		
	Sevilla	Spanish-style	n.dc	106.49 ± 0.26	545.42 ± 13.24	n.dr.		
	Sigoise	Spanish-style	1840.29 ± 49.27	35.84 ± 8.76	98.80 ± 19.8	n.dr.		
	Teffahi	Spanish-style	n.dc	24.38 ± 0.00	14.49 ± 1.49	n.dr.		
	Bouchouk	Spanish-style	n.dc	27.60 ± 4.23	100.60 ± 1.65	n.dr.		
	Azz Taz	Spanish-style	n.dc	24.35 ± 0.00	38.22 ± 0.00	n.dr.		
Tunisia	Chétoui	Spanish-style	307 ± 1.2	49 ± 0.2	3750 ± 8.3	n.dr.	HPLC-DAD	[30]
			480 ± 2	42 ± 0.02	2300 ± 9.3	n.dr.		
Turkey	Gemlik	Dry-salted black olives	231 ± 1	78 ± 0	221 ± 1	64 ± 0	LC-DAD-ESI-MS/MS	[31]

Non-determined (n.dr.), non-detected (n.dc.).

**Table 2:** Hydroxytyrosol (HT), tyrosol (TYR), hydroxytyrosol acetate (HT-AC), oleuropein-aglycone di-aldehyde (3,4-DHPEA-EDA), oleuropein-aglycone mono-aldehyde (3,4-DHPEA-EA), ligstroside-aglycone di-aldehyde (p-DHPEA-EDA), oleuropein (OLE) and secoiridoids derivative (S-DER) concentration in olive oil samples according to origin, variety, category, processing and analytical method.

Origin	Variety	Category	Concentration (mg/kg)									Method	References
			HT	TYR	HT-AC	3,4-DHPEA-EDA	3,4-DHPEA-EA	p-HPEA-EDA	p-HPEA-EA	OLE	S-DER		
Calabria Italy	Frantoio	EVOO	5.3 ± 0.9	5.4 ± 0.7	n.dr.	98.4 ± 3.5	27.5 ± 1.6	52.9 ± 1.7	4.4 ± 0.9	n.dr.	n.dr.	HPLC -DAD	[32]
Calabria Italy			4.6 ± 0.7	2.5 ± 0.3	1.9 ± 0.3	50.3 ± 2.8	22.5 ± 2.2	34.9 ± 1.7	3.0 ± 0.9	n.dr.	n.dr.		
Calabria Italy			1.2 ± 0.4	1.1 ± 0.2	n.d.	89.4 ± 2.9	19.0 ± 1.7	37.9 ± 1.6	n.d.	n.dr.	n.dr.		
Praia a mare, Calabria Italy			1.7 ± 0.1	1.7 ± 0.5	0.7 ± 0.1	56.0 ± 3.1	18.9 ± 1.9	34.6 ± 2.7	1.7 ± 0.9	n.dr.	n.dr.		
Italy	Coratina	VOO	1.97–7.1	5.7–16.4	n.dr.	65.1–147.7	94.6–268.9	89.4–153.7	25.4–120.8	n.dr.	n.dr.	IOC Extraction RP-HPLC-DAD	[33]
	Bosana		1.9–3.0	3.7–5.7	n.dr.	71.8–237.2	53.4–118.7	70.8–155.6	16.1–34.7	n.dr.	n.dr.		
	Semidana		1.3–4.8	2.7–9.2	n.dr.	4.5–90.8	11.6–66.2	17.0–57.5	5.5–17.2	n.dr.	n.dr.		
	Tonda di Cagliari		0.9–5.3	2.2–10.4	n.dr.	13.5–138.8	13.7–59.9	23.2–108.0	8.4–26.3	n.dr.	n.dr.		
Tuscany Italy	Multi- varietal	EVOO	161.5 ± 4.5	122.5 ± 3.5	n.dr.	n.dr.	n.dr.	n.dr.	n.dr.	n.dr.	n.dr.	Acid hydrolysis HPLC-DAD	[34]
Northern Italy	Frantoio	EVOO	0.9 ± 0.0	4.2 ± 0.6	1.3 ± 0.2	76.7 ± 55	7.9 ± 0.4	6.7 ± 0.9	n.dr.	18.5 ± 1.6	n.dr.	LLE HPLC-DAD-MS	[35]
			3.0 ± 0.2	4.3 ± 0.3	1.9 ± 0.2	62.2 ± 4.9	2.6 ± 0.3	2.2 ± 0.1	n.dr.	5.3 ± 0.3	n.dr.		
	Casaliva		0.7 ± 0.7	3.1 ± 0.4	1.8 ± 0.2	56.6 ± 4.7	3.2 ± 0.1	7.4 ± 0.4	n.dr.	6.6 ± 0.7	n.dr.		
			1.9 ± 0.3	4.6 ± 0.4	1.5 ± 0.2	55.0 ±0.8	1.4 ± 0.0	2.7 ± 0.2	n.dr.	1.8 ± 0.1	n.dr.		
	Organic Casaliva		2.3 ± 0.1	1.7 ± 0.1	1.8 ± 0.3	108.2 ± 9.6	9.2 ± 0.9	12.3 ± 1.2	n.dr.	12.8 ± 1.9	n.dr.		
			4.3 ± 0.3	4.9 ± 0.8	3. ± 0.23	98.9 ± 13.3	3.5 ± 0.1	7.3 ± 0.1	n.dr.	2.7 ± 0.2	n.dr.		
	Multi-varietal		6.4 ± 0.2	2.5 ± 0.4	3.4 ± 0.2	102.4 ± 4.6	5.5 ± 0.1	10.9 ± 1.0	n.dr.	10.0 ± 1.1	n.dr.		
			0.3 ± 0.0	1.1 ± 0.1	2.9 ± 0.2	54.6 ± 3.0	6.4 ± 0.6	0.9 ± 0.0	n.dr.	1.5 ± 0.1	n.dr.		
	Organic multi-varietal		11.4 ± 0.6	5.6 ± 0.6	1.7 ± 0.1	106.9 ± 12.6	8.8 ± 2.9	12.5 ± 0.6	n.dr.	15.4 ± 1.5	n.dr.		
			0.6 ± 0.0	2.8 ± 0.4	1.9 ± 0.3	89.4 ± 1.3	2.2 ± 0.1	14.4 ± 1.3	n.dr.	9.5 ± 0.7	n.dr.		
Salerno Italy	N/D (Cilento PDO)	VOO	37–41.3	23.8–34.6	n.dr.	n.dr.	19.9–24.9	n.dr.	23.8–35	120.4–140	n.dr.	RP-HPLC-DAD	[36]
Abruzzo Italy	Gentile	VOO	1.6–3.7	3.9–5.8	n.dr.	n.dr.	20.8–22.0	n.dr.	n.dr.	n.dr.	232.8–359.9	HPLC- DAD HPLC- MS	[37]
			0.8 ± 0.1	4.1 ± 0.2	n.dr.	n.dr.	15.2 ± 0.3	n.dr.	n.dr.	n.dr.	173.8 ± 3.0		
	Leccino		2.3 ± 0.1	6.1 ± 0.2	n.dr.	n.dr.	5.9 ± 0.1	n.dr.	n.dr.	n.dr.	91.0 ± 1.6		
			n.d.	4.0 ± 0.2	n.dr.	n.dr.	5.4 ± 0.2	n.dr.	n.dr.	n.dr.	120.1 ± 1.4		
	Dritta		2.0–2.8	7.4–7.9	n.dr.	n.dr.	14.9–30.9	n.dr.	n.dr.	n.dr.	189.9–299.1		
			3.5 ± 0.1	8.8 ± 0.24	n.dr.	n.dr.	11.3 ± 0.2	n.dr.	n.dr.	n.dr.	310.3 ± 5.3		
Southwest Spain	Arbequina	VOO	1.3 ± 0.4	1.9 ± 0.3	42.3 ± 3.6	75.5 ± 23.9	33.7 ± 1.9	59.9 ± 6.9	16.5 ± 0.9	n.dr.	n.dr.	SPE RP-HPLC-DAD	[38]
			0.4 ± 0.0	0.6 ± 0.0	36.8 ± 1.5	44.7 ± 1.1	104.4 ± 1.2	36.8 ± 0.2	9.7 ± 1.8	n.dr.	n.dr.		
	Carrasqueña		2.8 ± 0.8	3.5 ± 0.9	7.3 ± 0.3	141.1 ± 1.1	68.4 ± 14.9	73.5 ± 18.9	82.9 ± 6.9	n.dr.	n.dr.		
			0.6 ± 0.0	3.4 ± 0.0	6.9 ± 1.4	88.7 ± 10.5	95.2 ± 33.4	36.1 ± 3.1	14.3 ± 1.7	n.dr.	n.dr.		
	Corniche		2.0 ± 0.8	3.1 ± 1.9	n.d.	69.0 ± 14.7	61.2 ± 16.5	101.7 ± 21.5	12.4 ± 2.3	n.dr.	n.dr.		
			0.9 ± 0.3	2.1 ± 1.0	20.9 ± 2.5	150.5 ± 12.6	37.0 ± 9.3	116.9 ± 32.4	10.5 ± 3.4	n.dr.	n.dr.		
	Manzanilla Cacereña		1.5 ± 0.6	5.9 ± 1.4	n.dc.	56.0 ± 9.6	32.8 ± 9.8	58.2 ± 7.3	73.4 ± 0.9	n.dr.	n.dr.		
			0.7 ± 0.3	7.8 ± 1.5	n.dr.	40.6 ± 4.5	48.3 ± 5.9	59.4 ± 8.1	10.9 ± 3.1	n.dr.	n.dr.		
	Morisca		1.3 ± 0.3	3.0 ± 0.9	6.2 ± 10.0	70.6 ± 7.2	30.4 ± 4.1	41.4 ± 31.6	51.0 ± 1.2	n.dr.	n.dr.		
			1.4 ± 0.7	3.2 ± 1.4	43.3 ± 8.3	42.5 ± 5.3	18.9 ± 0.8	40.3 ± 3.6	23.9 ± 5.0	n.dr.	n.dr.		
	Picual		3.3 ± 1.2	5.7 ± 1.3	n.dc.	89.3 ± 15.6	73.3 ± 16.9	44.2 ± 3.5	39.8 ± 7.4	n.dr.	n.dr.		
			1.6 ± 0.8	3.5 ± 2.7	n.dc.	71.7 ± 3.9	46.9 ± 0.8	39.7 ± 7.5	68.8 ± 9.6	n.dr.	n.dr.		
	Verdial de Badajoz		1.4 ± 0.2	6.3 ± 0.6	n.dr.	143.8 ± 34.2	33.7 ± 5.7	166.4 ± 15.0	41.6 ± 11.8	n.dr.	n.dr.		
			0.8 ± 0.4	4.2 ± 1.1	n.dr.	71.6 ± 18.8	16.5 ± 1.7	123.5 ± 19.1	6.5 ± 0.5	n.dr.	n.dr.		
Jaén, Spain	Picual	VOO	2.8–7.8	4.9–9.9	n.dr.	221.4–849.7	29.5–929.2	19.5–248.9	24.4–344.2	n.dr.	n.dr.	LLE HPLC- DAD	[39]
			2.8–6.2	0.9–7.8	n.dr.	231.5–788.0	26.2–790.7	13.5–262.2	22.0–269.5	n.dr.	n.dr.		
Seville, Spain	Arbequina Hojiblanca Manzanilla Picual N/A	EVOO	50–200	40–180	n.dr.	n.dr.	n.dr.	n.dr.	n.dr.	n.dr.	n.dr.	Acid hydrolysis HPLC- UV-FL	[40]
	N/D	OO	5-20	5–30	n.dr.	n.dr.	n.dr.	n.dr.	n.dr.	n.dr.	n.dr.		
Spain	Picual-Arbequina blend	VOO	15.7 ± 0.9	9.1 ± 0.5	n.dr.	n.dr.	n.dr.	n.dr.	38.5 ± 3.8	n.dr.	n.dr.	SPE HPLC-ESI-TOF/MS	[41]
			14.3 ± 0.2	8.9 ± 0.3	n.dr.	n.dr.	n.dr.	n.dr.	44.8 ± 0.9	n.dr.	n.dr.	SPE HPLC-UV	
Catalonia Spain	Arbequina	VOO	2.5	3.0	1.6	152	68	20	42	n.dr.	26.4	LLE UPLC-MS/MS	[42]
Spain	Cornicabra	EVOO	0.9–2.8	1.0–2.3	n.dr.	396–770	136–301	228–498	39–138	n.dr.	n.dr.	SPE HPLC-DAD	[43]
Messenia, Greece	Koroneiki	EVOO	4.1 ± 0.1	0.4 ± 0.0	n.dr.	n.dr.	n.dr.	n.dr.	n.dr.	n.dr.	n.dr.	Selective ion monitoring GC/MS	[44]
			9.3 ± 0.1	0.4 ± 0.0	n.dr.	n.dr.	n.dr.	n.dr.	n.dr.	n.dr.	n.dr.		
			20.2 ± 1.0	0.4 ± 0.1	n.dr.	n.dr.	n.dr.	n.dr.	n.dr.	n.dr.	n.dr.		
Morocco	Arbequina	VOO	6.4 ± 2.7	11.6 ± 3.3	26.6 ± 32.7	n.dr.	55.4 ± 6.4	n.dr.	53.9 ± 4.4	n.dr.	n.dr.	LLE LC-ESI- IT-MS	[45]
	Arbosana		1.3 ± 1.9	3.3 ± 4.0	1.5 ± 1.3	n.dr.	21.0 ± 8.4	n.dr.	18.9 ± 2.2	n.dr.	n.dr.		
	Cornicabra		4.3 ± 0.4	6.6 ± 0.4	1.4 ± 0.1	n.dr.	96.9 ± 14.5	n.dr.	93.9 ± 6.4	n.dr.	n.dr.		
	Frantoio		0.8 ± 0.4	4.1 ± 0.6	0.3 ± 0.2	n.dr.	24.5 ± 14.6	n.dr.	34.8 ± 8.7	n.dr.	n.dr.		
	Hojiblanca		0.5 ± 0.3	5.4 ± 2.1	1.1 ± 0.8	n.dr.	35.8 ± 17.1	n.dr.	41.1 ± 18.1	n.dr.	n.dr.		
	Koroneiki		8.3 ± 2.6	7.5 ± 2.4	2.2 ± 0.3	n.dr.	93.6 ± 9.0	n.dr.	66.1 ± 15.8	n.dr.	n.dr.		
	Manzanilla		3.8 ± 3.3	10.8 ± 5.6	1.8 ± 1.5	n.dr.	54.0 ± 33.2	n.dr.	65.6 ± 37.1	n.dr.	n.dr.		
	P-Languedoc		2.2 ± 0.8	9.5 ± 1.2	0.8 ± 0.2	n.dr.	46.6 ± 10.9	n.dr.	46.3 ± 14.4	n.dr.	n.dr.		
	P-Marocaine		2.2 ± 1.7	8.5 ± 1.6	0.9 ± 0.3	n.dr.	35.2 ± 7.6	n.dr.	35.9 ± 3.7	n.dr.	n.dr.		
	Picual		4.8 ± 2.7	9.2 ± 2.0	2.2 ± 1.9	n.dr.	51.96 ± 26.81	n.dr.	47.4 ± 6.1	n.dr.	n.dr.		
	Dahbia		0.2 ± 0.0	1.7 ± 0.2	0.2 ± 0.0	n.dr.	26.1 ± 0.4	n.dr.	33.1 ± 0.9	n.dr.	n.dr.		
	Haouzia		3.7 ± 1.4	7.6 ± 2.0	0.5 ± 0.3	n.dr.	63.5 ± 12.7	n.dr.	51.1 ± 22.8	n.dr.	n.dr.		
	Menara		4.1 ± 2.7	11.4 ± 2.6	0.4 ± 0.2	n.dr.	41.6 ± 10.5	n.dr.	43.8 ± 5.3	n.dr.	n.dr.		
Algeria	Azeradj	EVOO	2.8–3.0	13.3–26.4	n.dr.	n.dr.	n.dr.	n.dr.	n.dr.	1.2–2.1	n.dr.	SPE HPLC- UV-vis	[46]
	Mekki		1.1–1.4	9.0–11.2	n.dr.	n.dr.	n.dr.	n.dr.	n.dr.	n.dc.	n.dr.		
	Neb djemel		4.4–7.3	15.1–17.2	n.dr.	n.dr.	n.dr.	n.dr.	n.dr.	n.dc.	n.dr.		
	Chemlal		3.3–4.2	13.6–19.2	n.dr.	n.dr.	n.dr.	n.dr.	n.dr.	0.0–4.4	n.dr.		
	Hamra		1.1–4.1	9.9–20.7	n.dr.	n.dr.	n.dr.	n.dr.	n.dr.	8.3–13.1	n.dr.		
	Blanquette de Guelma		4.8–8.9	19.5–21.6	n.dr.	n.dr.	n.dr.	n.dr.	n.dr.	1.3–2.2	n.dr.		
	Limli		2.9–3.2	13.1–18.2	n.dr.	n.dr.	n.dr.	n.dr.	n.dr.	n.dr.	n.dr.		
	Aberkane1		1.1–1.2	16.7–18.5	n.dr.	n.dr.	n.dr.	n.dr.	n.dr.	n.dr.	n.dr.		
	Aimell		2.2–6.3	18.0–18.3	n.dr.	n.dr.	n.dr.	n.dr.	n.dr.	n.dr.	n.dr.		
	Rougette de la mitidja		4.1–4.6	14.6–19.6	n.dr.	n.dr.	n.dr.	n.dr.	n.dr.	1.2–1.5	n.dr.		
	Aghenaou		1.7–2.4	14.0–18.1	n.dr.	n.dr.	n.dr.	n.dr.	n.dr.	n.dc.	n.dr.		
	Boughenfas		2.6–2.8	16.6–26.4	n.dr.	n.dr.	n.dr.	n.dr.	n.dr.	1.6–2.5	n.dr.		
	Bouichret		3.1–4.2	13.5–14.6	n.dr.	n.dr.	n.dr.	n.dr.	n.dr.	n.dc.	n.dr.		
	Aghenfas		3.2–3.9	18.4–25.1	n.dr.	n.dr.	n.dr.	n.dr.	n.dr.	0.8–5.2	n.dr.		
	Bouchouk de Guergour		3.8–4.2	14.5–20.2	n.dr.	n.dr.	n.dr.	n.dr.	n.dr.	0.3–0.5	n.dr.		
	X-Aghenfas		3.5–8.7	13.9–36.3	n.dr.	n.dr.	n.dr.	n.dr.	n.dr.	n.dc.	n.dr.		
	Bounguergueb		1.8–3.5	13.9–14.0	n.dr.	n.dr.	n.dr.	n.dr.	n.dr.	n.dc.	n.dr.		
	Ronde de miliana		0.0–1.5	16.6–20.2	n.dr.	n.dr.	n.dr.	n.dr.	n.dr.	1.0–1.5	n.dr.		
	Sigoise		1.4–2.6	22.4–27.9	n.dr.	n.dr.	n.dr.	n.dr.	n.dr.	0.2–0.4	n.dr.		
	Grosse du Hamma		9.67-14.93	28.1–32.9	n.dr.	n.dr.	n.dr.	n.dr.	n.dr.	0.4–0.9	n.dr.		
	Rougette de Guelma		3.3–3.7	14.0–18.5	n.dr.	n.dr.	n.dr.	n.dr.	n.dr.	0.7–1.4	n.dr.		
Tunisia	Oueslati	EVOO	3.8–7.2	1.9–3.1	0.3–1.3	n.dr.	222.6–537.8	n.dr.	2.9–19.4	n.dr.	n.dr.	RRLC- ESI- TOF-MS	[47]
Hatay Turkey	Halhali	VOO	5.5 ± 0.0	10.3 ± 0.2	n.dr.	n.dr.	n.dr.	n.dr.	n.dr.	n.dr.	n.dr.	LLE HPLC- DAD	[48]
		EVOO	5.2 ± 0.9	14.8 ± 0.4	n.dr.	n.dr.	n.dr.	n.dr.	n.dr.	n.dr.	n.dr.		
Balikesir Turkey	Ayvalik	VOO	0.1–0.8	0.7–1.1	n.dr.	n.dr.	n.dr.	n.dr.	n.dr.	n.dr.	n.dr.	HPLC- DAD	[49]
	Domat		0.0–1.2	0.2–0.9	n.dr.	n.dr.	n.dr.	n.dr.	n.dr.	n.dr.	n.dr.		
	Gemlik		0.2–0.4	0.5–1.6	n.dr.	n.dr.	n.dr.	n.dr.	n.dr.	n.dr.	n.dr.		

Non-determined (n.dr.), non-detected (n.dc.), non-declared (N/D).

**Table 3:** Tyrosol (TYR) and hydroxytyrosol (HT) content in wine according to origin, variety, and method of analysis.

Origin	Variety	Year	Concentration (mg/L)		Method	References
			TYR	HT		
France	Not specified		n.dr.	0.0092	LC-MS/MS	[50]
Australia		-		0.0054		
China				0.0056		
China				0.000071		
Salento, Italy	Negroamaro (Red wine)	-	n.dr.	2.3 ± 0.8 mg/kg	HPLC	[51]
	Primitivo (Red wine)	-		2.7 ± 0.7 mg/kg		
Croatia	Cabernet Sauvignon (Red wine)	2013	44.5	2.3	UV/VIS-HPLC	[52]
		2014	29.8	2.1		
		2015	46.4	1.746		
	Merlot (Red wine)	2013	40.7	2.7		
		2014	41.5	3.2		
		2015	36.9	2.6		
	Plavac mali (Red wine)	2013	41.3	2.9		
		2014	31.1	2.4		
		2015	48.3	2.6		
	Teran (Red wines)	2013	40.6	3.7		
		2014	21.6	4.0		
		2015	36.5	3.4		
Jerez de la Frontera, Spain	Corredera (White wine)	-	n.dr.	0.173	HPLC	[53]
	Moscatel (White wine)			0.159		
	Chardonnay (White wine)			0.167		
	Sauvignon Blanc (White wine)			0.288		
	Palomino Fino (White wine)			0.089		
	Vijiriega (White wine)			0.238		
Spain	Bach Viña Extrísimo (White wine)	2016	10.4	1.3	LC/MS-MS	[54]
Girona, Spain	Jardins Negre (Red wine)	2017	25.30	1.80	LC/MS-MS	[55]
Italy	Greco di Tufo (White wine)	1998	1.1	2.7	HPLC	[56]
	Verdicchio (White wine)	1998	3.0	1.6		
	Pinot Grigio (White wine)	1997	2.3	1.9		
	Blended	1998	5	6.1		
		1996	6	5.9		
	Barbera (Red wine)	-	5.9	9.6		
	Montepulciano (Red wine)	1998	5.9	0.5		
Italy	White wine		1.42–2.34	1.79–2.00	GC-MS	[57]
	Red wine		3.61–4.80	3.81–4.37		
Jerez de la Frontera, Spain	Tempranillo (Red wine)	2009	20.51–22.76	0.82–1.84	HPLC-FD	[58]
	Blasco (Red wine)		27.38	1.55		
	Cabernet Sauvignon (Red wine)		31.95–32.57	5.02–3.63		
	Petit Verdot (Red wine)		40.59–38.50	4.12–2.33		
	Syrah (Red Wine)	2010	40.98–34.11	1.11–0.82		
	Merlot (Red Wine)		44.46	1.77		
	Tintilla de Rota (Red Wine)		28.91–30.97	2.66–1.65		
	Melonera (Red Wine)		35.31–36.86	0.53–0.45		
	Tempranillo (Red Wine)		25.03–44.26	1.78		
	Vitis silvestris (Red Wine)		20.38–40.20	0.28–3.09		
	Palomino negro (Red Wine)		29.57	1.10		
	Rome (Red Wine)		35.41–35.57	1.67–1.86		
	Garnacha (Red Wine)		26.73–30.15	1.02–1.28		

Non-determined (n.dr.).

**Table 4:** Dietary surveys for the adult population in the European countries used in this study (EFSA Comprehensive European Food Consumption Database).

Country	Name of the Dietary Survey	Period of Survey	Nº of Subjects
Austria	Austrian Study on Nutritional Status 2010–2012—Adults (ASNS–Adults)	2010–2012	615
Belgium	Belgian National Food consumption survey (NATIONAL-FCS-2014)	2014–2015	2278
Croatia	Croatian food consumption survey on adults (NIPNOP-HAH-2011-2012)	2011–2012	2000
Czech Republic	Czech National Food Consumption Survey (SISP04)	2003–2004	1666
Cyprus	National dietary survey of the adult population of Cyprus (CY 2014-2017-LOT2)	2014–2017	812
Denmark	The Danish National Dietary survey 2005–2008 (DANSDA 2005-08)	2005–2008	1739
Estonia	National Dietary Survey among 11–74 years old individuals in Estonia (DIET-2014-EST-A)	2013–2015	2124
Finland	National FINDIET 2012 Survey (FINDIET2012)	2012	1295
France	The French national dietary survey (INCA3)	2014–2015	1773
Germany	National Nutrition Survey II	2007	10,419
Greece	The EFSA-funded collection of dietary or related data in the general population aged 10–74 years in Greece (GR-EFSA-LOT2 2014-2015)	2014–2016	791
Hungary	National Repr Surv (NATIONAL REPR SURV)	2003	1074
Ireland	National Adult Nutrition Survey (NANS 2012)	2008–2010	1274
Italy	Italian National Food Consumption Survey (INRAN SCAI 2005-06)	2005–2006	2313
Latvia	Latvian National Dietary survey (LATVIA_2014)	2012–2015	1080
Netherlands	Dutch National food consumption survey 2012–2016 (FCS2016_CORE)	2012–2017	4313
Portugal	National Food, Nutrition and Physical Activity Survey of the Portuguese general population (IAN.AF 2015-2016)	2015–2016	3102
Romania	Dietary Pilot Adults	2012	1254
Slovenia	Slovenian national food consumption survey (SI. MENU-2018)	2017–2018	2119
Spain	Spanish National dietary survey in adults, elderly, and pregnant woman (ENALIA2)	2013–2015	669
Sweden	Swedish National Dietary Survey—Riksmaten adults 2010–11 (RIKSMATEN 2010)	2010–2011	1430
United Kingdom	National Diet and Nutrition Survey (NDNS)	2000–2001	1724

**Table 5:** Summary of the health benefits and mechanism of action of HT.

Health Benefit	Mechanism of Action	References
Anti-inflammatory	Inhibition of LPS mediated expression of TNF-α and IL-1β ↓ Expression of NADPH oxidase and MAPKs ↓ Inflammasome	[5,6]
Neuroprotective	Inhibition of the formation of α-synuclein and β-amyloid fibrils	[7,8,9]
Antiangiogenic	Inhibition of VEGF receptor-2 activation	[10]
Pro-apoptotic	↓ Proliferation of MCF-7	[13,14]
Anti-diabetic	Insulin-like effect on target cells	[61,62]
Antioxidant	Blood lipids’ protection from oxidative stress	[15]

**Table 6:** Extra Virgin Olive Oil (EVOO)/ Virgin Olive Oil (VOO), table olives and wine (according to levels 3–6 of the FoodEx2 classification system for the adult population) consumption and free hydroxytyrosol daily intake for adult consumers (18–64 years) based on the EFSA Comprehensive European Food Consumption Database.

Foods under Study	Food Consumption	Free Hydroxytyrosol Daily Intake from Foods under Study	HT from Oleuropein and Aglycone
	Range of Means among EU Surveys (g/day)	Range of Means (mg/day)	Range of Means (mg/day)
EVOO/VOO	0–33.78	0–0.18	0–1.08
Table olives	0.02–3.41	0.010–2.14	0.03–6.42
Wine	10.65–89.08	0.022–0.186	-

**Table 7:** Food ingredients and future trends.

Food	Quantity	Origin	References
Yogurt	5 mg HT/120 g (1 unit = 120 g)	Olive Pomace Liquid-enriched powder (LOPP) and pulp-enriched powder (POPP) obtained from olive pomace	[93]
Fuet (Dried cured sausage)	200 mg/kg	Synthetic	[94]
Fuet (Dried cured sausage)	200 mg/kg	Olive vegetation waters of olive	[94]
Cookies	5.25 mg/30 g	Olive oil wastewaters	[95]
Smoothies	5.78 mg HT + OLE/100 g	Olive leaf extracts	[96]
Chicken sausage	50 mg/kg	Olive water/olive leaves	[97]
Blood orange juice	28.8–57.6 mg/L	Olive mill wastewater	[98]

## Data Availability

Data will be available from the corresponding author on reasonable request.
